# *SlGAD2* is the target of SlTHM27, positively regulates cold tolerance by mediating anthocyanin biosynthesis in tomato

**DOI:** 10.1093/hr/uhae096

**Published:** 2024-04-04

**Authors:** Jingrong Wang, Yong Zhang, Junzheng Wang, Abid Khan, Zheng Kang, Yongbo Ma, Jiarui Zhang, Haoran Dang, Tianlai Li, Xiaohui Hu

**Affiliations:** College of Horticulture, Northwest A&F University, Yangling, Shaanxi, 712100, China; Key Laboratory of Protected Horticultural Engineering in Northwest, Ministry of Agriculture and Rural Affairs, Yangling, Shaanxi, 712100, China; Shaanxi Protected Agriculture Engineering Technology Research Centre, Yangling, Shaanxi, 712100, China; College of Horticulture, Northwest A&F University, Yangling, Shaanxi, 712100, China; Key Laboratory of Protected Horticultural Engineering in Northwest, Ministry of Agriculture and Rural Affairs, Yangling, Shaanxi, 712100, China; Shaanxi Protected Agriculture Engineering Technology Research Centre, Yangling, Shaanxi, 712100, China; College of Horticulture, Northwest A&F University, Yangling, Shaanxi, 712100, China; Key Laboratory of Protected Horticultural Engineering in Northwest, Ministry of Agriculture and Rural Affairs, Yangling, Shaanxi, 712100, China; Shaanxi Protected Agriculture Engineering Technology Research Centre, Yangling, Shaanxi, 712100, China; Department of Horticulture, The University of Haripur, Haripur 22620, Pakistan; College of Horticulture, Northwest A&F University, Yangling, Shaanxi, 712100, China; Key Laboratory of Protected Horticultural Engineering in Northwest, Ministry of Agriculture and Rural Affairs, Yangling, Shaanxi, 712100, China; Shaanxi Protected Agriculture Engineering Technology Research Centre, Yangling, Shaanxi, 712100, China; College of Horticulture, Northwest A&F University, Yangling, Shaanxi, 712100, China; Key Laboratory of Protected Horticultural Engineering in Northwest, Ministry of Agriculture and Rural Affairs, Yangling, Shaanxi, 712100, China; Shaanxi Protected Agriculture Engineering Technology Research Centre, Yangling, Shaanxi, 712100, China; College of Horticulture, Northwest A&F University, Yangling, Shaanxi, 712100, China; Key Laboratory of Protected Horticultural Engineering in Northwest, Ministry of Agriculture and Rural Affairs, Yangling, Shaanxi, 712100, China; Shaanxi Protected Agriculture Engineering Technology Research Centre, Yangling, Shaanxi, 712100, China; College of Horticulture, Northwest A&F University, Yangling, Shaanxi, 712100, China; Key Laboratory of Protected Horticultural Engineering in Northwest, Ministry of Agriculture and Rural Affairs, Yangling, Shaanxi, 712100, China; Shaanxi Protected Agriculture Engineering Technology Research Centre, Yangling, Shaanxi, 712100, China; College of Horticulture, Shenyang Agricultural University, Shenyang, Liaoning 110866, China; College of Horticulture, Northwest A&F University, Yangling, Shaanxi, 712100, China; Key Laboratory of Protected Horticultural Engineering in Northwest, Ministry of Agriculture and Rural Affairs, Yangling, Shaanxi, 712100, China; Shaanxi Protected Agriculture Engineering Technology Research Centre, Yangling, Shaanxi, 712100, China

## Abstract

Cold stress significantly limits the yield and quality of tomato. Deciphering the key genes related to cold tolerance is important for selecting and breeding superior cold-tolerant varieties. γ-aminobutyric acid (GABA) responds to various types of stress by rapidly accumulating in plant. In this study, glutamic acid decarboxylase (GAD2) was a positive regulator to enhance cold stress tolerance of tomato. Overexpression of *SlGAD2* decreased the extent of cytoplasmic membrane damage and increased the endogenous GABA content, antioxidant enzyme activities, and reactive oxygen species (ROS) scavenging capacity in response to cold stress, whereas *Slgad2* mutant plants showed the opposite trend. In addition, *SlGAD2* induced anthocyanin biosynthesis in response to cold stress by increasing the content of endogenous GABA. Further study revealed that *SlGAD2* expression was negatively regulated by the transcription factor SlTHM27. However, the transcript levels of *SlTHM27* were repressed under cold stress. Antioxidant enzyme activities, *SlGAD2* transcript levels, GABA and anthocyanin contents were significantly increased in *Slthm27* mutant plants. Further, our study demonstrated that SlTHM27 decreases *SlGAD2*-promoted cold resistance in tomato by repressing *SlGAD2* transcription. Overall, our results showed that the SlTHM27-*SlGAD2* model regulates the cold tolerance in tomato by regulating GABA and anthocyanin.

## Introduction

Abiotic stresses such as salt, heat, cold, and drought, are among the major factors contributing to the decline in global crop yields and quality [1, 2]. Although plants have evolved with the ability to resist environmental stresses, the frequency and intensity of stresses encountered by plants have increased in recent years due to climate change [3, 4]. Among these stresses, low temperature is an unavoidable environmental factor that limits agricultural productivity [4]. Below 12°C, high levels of oxidative metabolites accumulate in the plant, affecting the protein and DNA structure, damaging the biofilm and plant tissues, and consequently inhibit the plant growth [5, 6]. Various researches have indicated that plants can scavenge ROS generated by enzymatic antioxidant systems (SOD, POD, CAT, APX, etc.) and non-enzymatic antioxidant systems (ASH, GSH, carotenoids, and flavonoids, etc.) under cold stress [6, 7]. Flavonoids are a class of highly biologically active plant secondary metabolites that have surpassed the performance of some common antioxidants [7]. As active oxygen scavengers, flavonoids reduce free radical damage to plant cells under unfavorable conditions by localizing and neutralizing free radicals [8].

Anthocyanins are a class of flavonoids. They not only impart vibrant colors to nutritive tissues such as flowers, leaves, and fruits of the plants, but also act as strong antioxidants for ROS scavenging and against microorganisms in defense reactions [9, 10]. Anthocyanin biosynthesis includes a series of enzymes such as chalcone isomerase (CHI), chalcone synthase (CHS), flavonoid 3-hydroxylase (F3H), dihydroflavonol 4-reductase (DFR), and UDP-glycosidic flavonoid transferase (UFGT) [9, 11]. Several transcription factors (TFs) have also been found to regulate the expression of these anthocyanin-synthesizing genes, such as SlANT1 and SlAN2 of the MYB family, SlGL3 and SlTT8 of the bHLH class, HY5 and BBX20 [12–16]. More and more evidence suggested that low temperature induces the expression of anthocyanin synthesizing genes, which in turn boosts the production of anthocyanins, and at the same time, the anthocyanin accumulation can also improve low temperature tolerance of the plants [17, 18]. Crifò *et al.* also discovered that low temperatures promoted anthocyanin accumulation in blood oranges [19]. *MdMYB308L* improved the cold stress tolerance of apple through anthocyanin accumulation [20].

Gamma-aminobutyric acid (GABA) acts a key factor in the regulation of plant growth, carbon/nitrogen balance, gene expression, ion homeostasis, and oxidative homeostasis under abiotic stresses [21–23]. Pretreatment with GABA has increased the cold tolerance of tomato and peach fruits [24]. Exogenous GABA significantly up-regulated the expression of WRKY75 and MYB13, and improved the tolerance of *Agrostis stolonifera* L. to drought [25]. Liu *et al.* discovered that GABA is an effective osmotic agent to reduce reactive oxygen species production in tobacco (*Nictiana tabacum* L.) under water stress [26]. In addition, GABA can also alleviate plant damage caused by stresses such as high temperature [22, 25], low temperature [27], salt [28], and heavy metals [29] through rapid accumulation. Of course, GABA is also a signaling molecule that activates the phenylalanine pathway and enriches flavonoids, including anthocyanins [30].

In plants, glutamic acid decarboxylase (GAD) is the rate-limiting enzyme for GABA synthesis by catalyzing the irreversible synthesis of GABA from glutamic acid (Glu) [28]. The expression of *GAD1* in mulberry leaves is induced by NaCl which promotes the synthesis of GABA, and consequently enhances the salt tolerance of mulberry leaves [31]. The increased transcript levels of *CiGAD1* and *CiGAD2* promoted the accumulation of GABA, and improved the salt stress resistance in mallow [2]. Cold stress significantly increased the GABA content in quinoa [32]. These studies demonstrated that *GAD* is the most sensitive gene for GABA synthesis under abiotic stress. Globally, tomato (*Solanum lycopersicum* L.) is a widely grown economic crop with high nutritional value [33]. Because tomato originates from the tropics, the low temperatures negatively affect its growth, yield, and quality [33]. Although a lot of studies have been done on how low temperatures alter anthocyanin biosynthesis, the regulation network of GABA content and anthocyanin in cold-stressed tomato remains unclear.

Our study revealed that exogenous GABA (55 mM) significantly improved the low temperature tolerance of tomato. In addition, *SlGAD2* was significantly induced by analysing the transcript levels of GABA synthesis-related genes (*SlGAD1–5*) at low temperature. We also identified SlTHM27, a R2R3 MYB-like TF, responds to cold stress by repressing the expression of *SlGAD2*. Interestingly, endogenous GABA increased anthocyanin accumulation under cold stress. Taken together, we revealed a novel pathway that is SlTHM27-*SlGAD2* to regulate cold stress, which might have potential applications in molecular breeding.

## Results

### Cold induces GABA accumulation and exogenous GABA enhances cold tolerance in tomato

**Figure 1:**
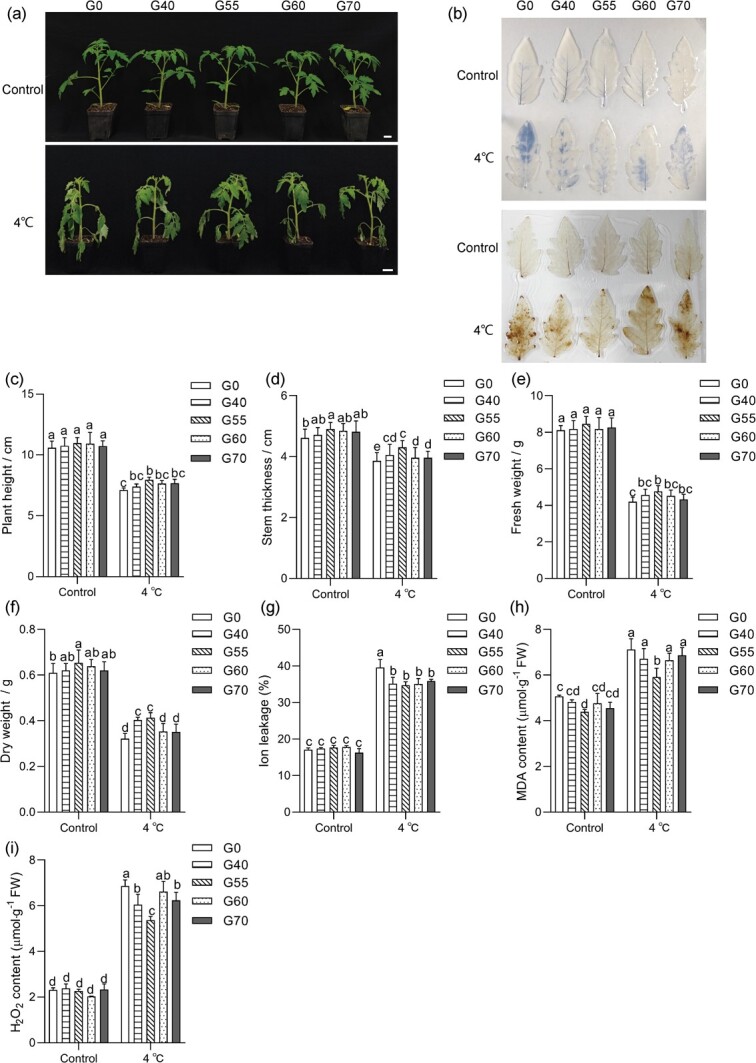
Exogenous γ-aminobutyric acid (GABA) on the cold tolerance of tomato seedlings. (**a**) Phenotypic changes in cold tolerance of tomato seedlings treated with different concentrations of GABA. Bar, 2.5 cm. G0, G40, G55, G60, and G70 mean exogenous sprays of 0 mM, 40 mM, 55 mM, 60 mM, and 70 mM of GABA, respectively. (**b**) Nitroblue tetrazolium (NBT) and diaminobenzidine (DAB) staining of leaves with water or GABA-treated plants under control and cold treatment (4°C) for 4 days. (**c**–**f**) Changes in plant height (**c**), stem thickness (**d**), fresh (**e**), and dry weight (f) of water or GABA-treated plants under control and cold treatment (4°C) for 4 days. (**g**–**i**) ion leakage (**g**), MDA (**h**), and H_2_O_2_ (**i**) of water or GABA-treated plants measured before or after cold treatment. Values represent the average of six (**c**–**f**) or four (**g**–**i**) independent measurements, and error bars represent standard errors. Different letters of the columns indicate significant differences (*P* < 0.05).

Due to the lack of knowledge about GABA accumulation in tomato seedlings under cold stress, we measured endogenous GABA levels in tomato seedlings at 4°C. GABA levels accumulated significantly with the duration of cold treatment and peaked at 48 h (Fig. S1, see online supplementary material). To investigate the role of GABA in cold response, the different concentrations of GABA were applied to wild-type (WT) tomato seedlings. Under normal environmental conditions, there was no significant difference in plant height, fresh weight and dry weight of tomato seedlings by exogenous GABA supply compared to the G0 (0 mM GABA) treatment (Fig. 1a, c–f). However, under low temperature treatment, spraying 55 mM GABA resulted in better seedling status compared to the other concentration treatments (Fig. 1a). Further analysis showed that exogenous spraying of 55 mM GABA (G55) significantly increased cold-stressed tomato seedings height, stem thickness, fresh and dry weight (Fig. 1c–f). Ion leakage reflects the extent of stress induced damage to plasma membrane [34]. Compared with the control, low temperature treatment significantly increased the ion leakage and MDA content in tomato seedling. Exogenous spraying of 55 mM GABA resulted in a significant decrease in ion leakage and MDA levels of the seedlings at low temperature (Fig. 1g and h). At the same time, we found that under low temperature stress, tomato seedlings accumulated a large amount of H_2_O_2_ whereas its accumulation in the GABA (55 mM) treated seedlings was significantly reduced (Fig. 1b and i). In conclusion, cold promoted the accumulation of GABA in tomato, while exogenous 55 mM GABA attenuated the cold-induced injury.

### *SlGAD2* is induced by cold stress in tomato

**Figure 2:**
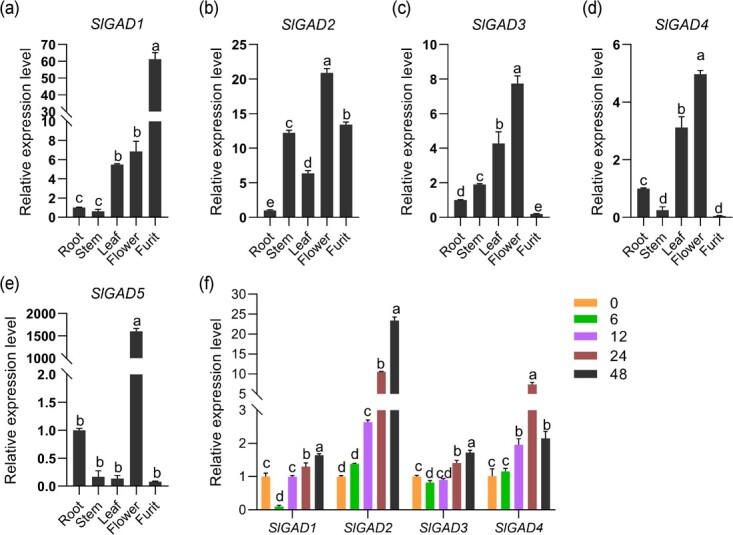
*SlGAD2* was significantly induced by cold stress. (**a**–**e**) Changes in transcript levels of the GABA synthesis-related genes *SlGAD1* (**a**), *SlGAD2* (**b**), *SlGAD3* (**c**), *SlGAD4* (**d**), and *SlGAD5* (**e**) in roots, stems, leaves, flowers, and ripe fruits of tomato. (**f**) Changes in relative expression of *SlGAD1–5* in leaves of tomato under cold treatment. 0 h in all genes be set to 1. Different letters of the columns indicate significant differences (*P* < 0.05).

To reveal the genes involved in cold-induced GABA accumulation, we cloned five *GAD* genes with 86.28% sequence alignment identity and high homology in the conserved regions (Fig. S2, see online supplementary material). Under normal environmental conditions, the expression of five *GAD* homologs was analysed in different tissues of WT. There were significant differences in the transcript levels of the five *GADs* in different tomato tissue. Among them, *SlGAD1* was heavily induced in leaves, flowers and fruits (Fig. 2a); *SlGAD5* was more highly expressed in flowers than in other tissues (Fig. 2e). *SlGAD3* and *SlGAD4* were significantly induced in leaves and flowers (Fig. 2a–d). *SlGAD2* was highly expressed in all tissues (Fig. 2b).

Four *GADs* (*SlGAD1*, *SlGAD2*, *SlGAD3*, and *SlGAD4*) with high transcript levels in tomato leaves were explored in response to cold stress. RT-qPCR results showed that all four selected *GADs* were induced under low temperature treatment, but compared with the other three *SlGADs*, only *SlGAD2* was most significantly induced and continuously upregulated (Fig. 2f).

### *SlGAD2* positively regulates anthocyanin synthesis and antioxidant enzyme activities to enhance tomato cold tolerance

To explore the role of *SlGAD2* in low temperature tolerance, we obtained two *SlGAD2* overexpressing transgenic lines (*SlGAD2* OE#4 and *SlGAD2* OE#5) in the ‘Ailsa Craig’ tomato background. It was also confirmed that *SlGAD2* expression was significantly increased in both transgenic lines (Fig. S3, see online supplementary material). After 4 days of exposure to low temperature (4°C), the *SlGAD2* OE#4 and *SlGAD2* OE#5 exhibited a cold tolerance phenotype as compared to WT (Fig. S4a, see online supplementary material). Meanwhile, *SlGAD2* overexpression plants had higher GABA level than WT under normal conditions, whereas low temperature increased GABA accumulation, especially in the *SlGAD2* overexpression plants (Fig. S4b, see online supplementary material). Under low temperature treatment, the *SlGAD2* overexpressed lines had lower ion leakage, MDA content, H_2_O_2_ and O_2_^−^ accumulation as compared to WT (Fig. S4c–f, see online supplementary material). In addition, *SlGAD2*-overexpressing lines also have higher SOD, POD, and CAT activities than WT (Fig. S4g–i, see online supplementary material), which is consistent with their phenotype of higher cold tolerance.

Surprisingly, under low-temperature stress we found pigmentation near the veins in the leaves of *SlGAD2* overexpressed plants (Fig. S5a, see online supplementary material). Based on quantitative analysis of anthocyanin levels, anthocyanin levels in the leaves of overexpressed *SlGAD2* plants were higher than those of WT under normal conditions and were especially more pronounced under cold stress (Fig. S5b, c, see online supplementary material). The transcript levels of genes involved in anthocyanin synthesis were also analysed. The RT-qPCR results showed that low temperature treatment induced the transcription of *SlCHS*, *SlF3H*, *SlDFR*, and *SlUFGT*, and it was more so in the *SlGAD2* overexpressed plants (Fig. S5d–g, see online supplementary material). In summary, overexpression of *SlGAD2* significantly increased antioxidant enzyme activities and anthocyanin level in transgenic plants, leading to improved cold tolerance.

To further verify the relationship between GABA levels and anthocyanin accumulation, we examined the endogenous GABA and anthocyanin levels in WT tomato leaves after exogenous application of GABA (55 mM). The results showed exogenous spraying of GABA significantly increased the anthocyanin content compared with the control, and this difference was more pronounced under cold stress (Fig. S6a, see online supplementary material). In addition, exogenous sprayed GABA significantly increased the anthocyanin content of tomato leaves (Fig. S6b, see online supplementary material). Thus, the accumulation of endogenous GABA helped to promote the increase of anthocyanins in tomato leaves.

**Figure 3:**
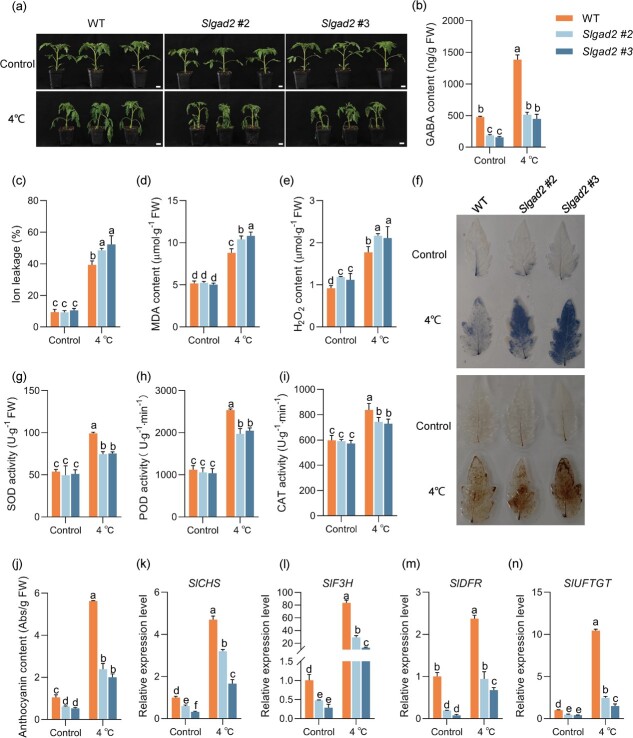
*Slgad2* mutant plants are more sensitive to cold stress. (**a**) Phenotypic changes in WT and *Slgad2* mutant plants treated at low temperature (4°C) for 4 days. Bar, 2.5 cm. (**b**) Endogenous GABA content tomato leaves shown in (**a**). (**c**–**e**) Electrolyte leakage (**c**), MDA content (**d**) and H_2_O_2_ content (**e**) of *Slgad2* mutant plants under control and cold stress (4°C). (**f**) NBT and DAB staining of leaves from WT and *Slgad2* mutant lines under control and cold stress (4°C). (**g**–**i**) SOD activity (**g**), POD activity (**h**) and CAT activity (**i**) in *Slgad2* mutant plants under control and cold stress. Plants were exposed to cold stress for 0 and 4 days, and leaves were collected for the measurements. (**j**) Anthocyanin content of *Slgad2* mutant plants under control and cold stress (4°C). (**k**–**n**) Relative expression levels of *SlCHS*, *SlF3H*, *SlDFR*, and *SlUFGT* in *Slgad2* mutant plants under cold treatment for 4 days. *SlACTIN* was used as an internal control. Different letters of the columns indicate significant differences (*P* < 0.05).

To further confirm that the cold-tolerant phenotype of *SlGAD2* OE is caused by enhanced *SlGAD2* function, we constructed *SlGAD2* mutants using CRISPR-Cas9 mediated targeting mutagenesis in the ‘AC’ background and selected two mutants without the CRISPR-Cas9 transgene for low temperature treatment (Fig. S7, see online supplementary material). Under cold stress, *Slgad2* mutant plants exhibited a cold-sensitive phenotype compared to WT (Fig. 3a). Meanwhile, the GABA content of *Slgad2* mutant plants was much lower than that of WT both under normal culture conditions and cold treatment (Fig. 3b). Compared with WT, *Slgad2* mutant plants had higher ion leakage level, MDA, and H_2_O_2_ content and lower SOD, POD, and CAT activities under cold stress (Fig. 3c–i).

Anthocyanin level of *Slgad2* mutant plants were reduced compared to the WT. This difference was more pronounced under cold stress (Fig. 3j). Similarly, the transcript levels of *SlCHS*, *SlF3H*, *SlDFR*, and *SlUFGT* were reduced in *Slgad2* mutant plants, especially more significantly under low temperature conditions (Fig. 3k–n). Analysis of these data further supports that *SlGAD2* plays a positive role in tomato cold tolerance and anthocyanin accumulation.

### SlTHM27 is a transcription factor regulating *SlGAD2*

**Figure 4:**
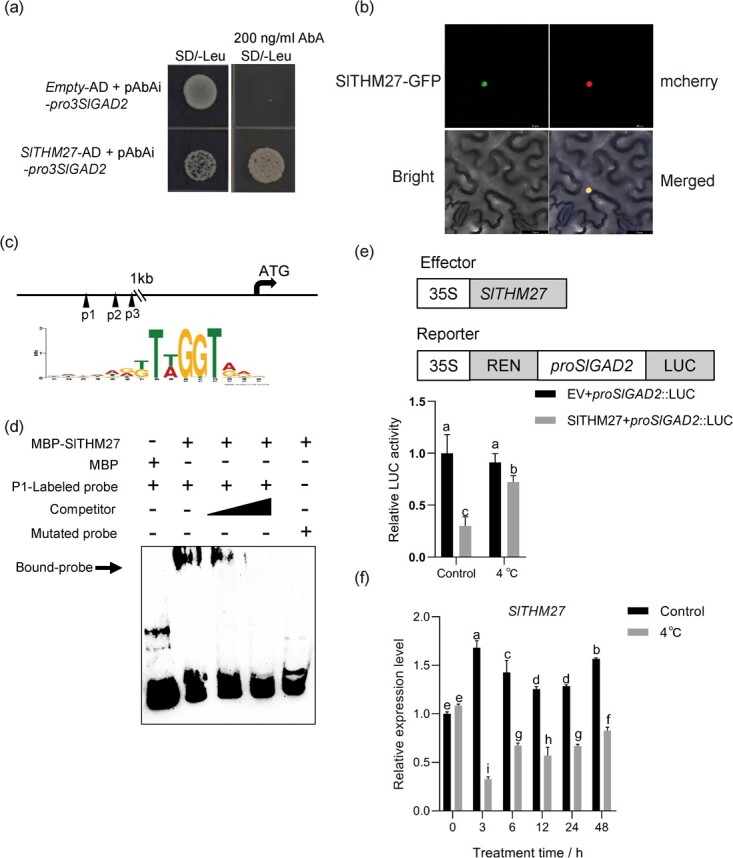
Interaction of SlTHM27 with *SlGAD2* promoter. (**a**) An interaction between SlTHM27 and promoters of *SlGAD2* by Y1H assays. Empty-AD as a control. (**b**) Subcellular localization of SlTHM27-GFP and NLS-mCherry in tobacco (*Nicotiana benthamiana*) leaves. Bar, 50 μM. (**c**) Schematic representation of the SlTHM27 binding element position on the *SlGAD2* promoter predicted by PlantTFDB. Black triangles represents the predicted positions of the binding elements. p1 represents the ‘TTAGGT’ binding element, p2 and p3 represent the ‘TTTGGT’ binding element. (**d**) SlTHM27-MBP was able to bind to the p1 site of the promoter in *SlGAD2* by EMSA analysis. (**e**) The inhibition of *SlGAD2* transcription by SlTHM27 was verified by assaying relative LUC/REN activity. The control used was the empty vector pGreen II 62-SK (EV). In cold treatment, infested tobacco was grown under normal conditions for 69 h and then in a growth chamber at 4°C for another 3 h, after which the leaves were collected. (**f**) *SlTHM27* relative expression changes before and after cold treatment. Different letters of the columns indicate significant differences (*P* < 0.05).

To determine the upstream transcription factor of *SlGAD2*, a tomato cDNA library was screened using Y1H with the *SlGAD2* promoter fragment as bait. SlTHM27 (Solyc10g055410.1), a protein of the R2R3 MYB family, has been screened and is a homologue of MdMYB16 with 66.67% sequence similarity (Fig. 4a; Fig. S8, see online supplementary material). SlTHM27 was only present in the nucleus according to subcellular localization results (Fig. 4b).

By searching the PlantTFDB database (http://planttfdb.gao-lab.org/prediction.php), we found that the *SlGAD2* promoter sequence was present with the SlTHM27 putative binding elements TTAGGT and TTTGGT motifs (Fig. 4c). Using an electrophoretic mobility shift assay (EMSA), we found that the SlTHM27-MBP fusion protein was able to bind to the TTAGGT element site but not the TTTGGT element in the *SlGAD2* promoter region (Fig. 4d; Fig. S9, see online supplementary material). Addition of a competing probe resulted in a decrease in binding strength. Mutation of ‘TTAGGT’ to ‘AAAAA’ significantly reduced the binding capacity (Fig. 4d). Thus, SlTHM27 binds directly to the promoter of *SlGAD2*.

To further characterize the role of SlTHM27 in gene activation, we performed a dual luciferase assay on tobacco leaves. A 1.5 kb *SlGAD2* promoter fragment driving a firefly LUC reporter construct was used to generate an effector construct using SlTHM27 (Fig. 4e). At 25°C, SlTHM27 significantly repressed the expression of *SlGAD2*, but this repression was significantly alleviated after low temperature treatment (Fig. 4e). The qRT-PCR results showed that the transcript levels of *SlTHM27* were all significantly higher than 0 h under normal conditions, while the transcript levels of *SlTHM27* were significantly suppressed under low temperature conditions, especially with the lowest expression level of *SlTHM27* at 3 h of low temperature treatment (Fig. 4f). Furthermore, we performed sequence comparison with homologous genes in Arabidopsis, apple and tomato and found that SlTHM27 has an EAR motif at its C-terminal end (Fig. S10, see online supplementary material). The EAR motif is a key feature of EAR-type transcriptional inhibitors [35].

### SlTHM27 negatively regulates anthocyanin biosynthesis and antioxidant enzyme activities to reduce tomato cold tolerance

**Figure 5:**
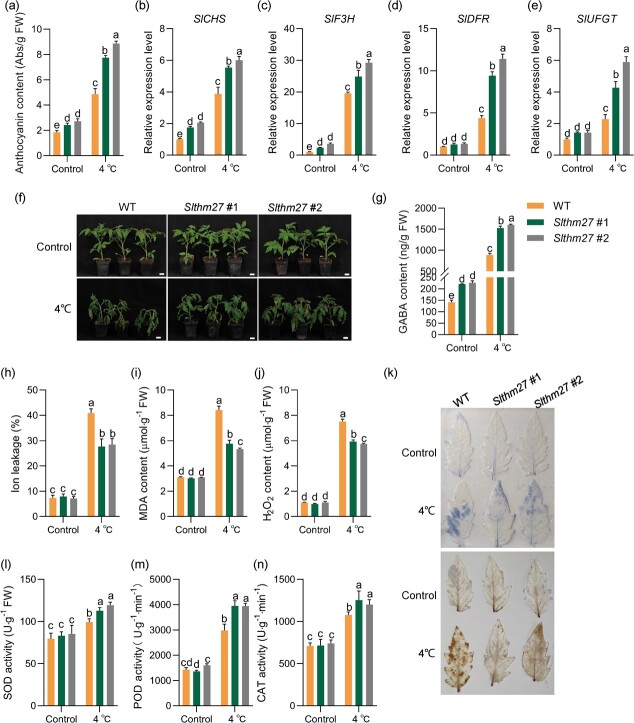
*SlTHM27* negatively regulates cold stress in tomato. (**a**) Anthocyanin content of *Slthm27* mutant plants under control and cold stress (4°C). (**b**–**e**) Relative expression levels of *SlCHS*, *SlF3H*, *SlDFR*, and *SlUFGT* under cold treatment in *Slthm27* mutant plants for 4 days. (**f**) Phenotypic changes in WT and *Slthm27* mutant plants treated at low temperature (4°C) for 4 days. Bar, 2.5 cm. (**g**) Endogenous GABA content tomato leaves shown in (**f**). (**h**–**j**) Electrolyte leakage (**h**), MDA content (**i**) and H_2_O_2_ content (**j**) of *Slthm27* mutant plants under control and cold stress (4°C). Plants were exposed to cold stress for 4 days, and leaves were collected for an ion leakage assay, MDA and H_2_O_2_ content measurement. (**k**) NBT and DAB staining of leaves from WT and *SlTHM27*-silenced lines under control and cold stress (4°C). (**l**–**n**) SOD activity (**l**), POD activity (**m**), and CAT activity (**n**) in *Slthm27* mutant plants under control and cold stress. Plants were exposed to cold stress for 0 and 4 days, and leaves were collected for the measurements. Different letters of the columns indicate significant differences (*P* < 0.05).

Because SlTHM27 has a high sequence similarity (66.67%) to MdMYB16, which was previously reported to negatively regulate anthocyanin synthesis in apple [35], we constructed the *SlTHM27* mutants using CRISPR-Cas9 mediated target mutagenesis (Fig. S11, see online supplementary material). We evaluated anthocyanin accumulation in tomato leaves of *Slthm27* mutant plants. Anthocyanin content measurements showed that *Slthm27* mutant plants accumulated significantly higher levels of anthocyanins than WT under both normal and low temperature treatments conditions (Fig. 5a). RT-qPCR results showed that *SlCHS*, *SlF3H*, *SlDFR*, and *SlUFGT* expression levels were higher in *Slthm27* mutant plants, and this difference was more significant under cold treatments (Fig. 5b–e). Studies have proven a positive correlation between anthocyanins and cold tolerance in plants [17]. We would like to further analyse the role of *SlTHM27* under cold stress in tomato.

We found no morphological differences between WT and *Slthm27* mutant plants (*Slthm27* #1 and *Slthm27* #2) under normal conditions. However, the *Slthm27* mutant plants showed a more cold-tolerant phenotype compared to WT under low temperature (Fig. 5f). Meanwhile, *Slthm27* mutant plants showed significantly lower ion leakage and MDA level than control, indicating less damage to membrane lipids compared to the WT under low temperature (Fig. 5h and i). Furthermore, the *Slthm27* mutant lines showed lower accumulation of H_2_O_2_, O_2_^−^ and higher SOD, POD, and CAT activities than WT under cold treatment (Fig. 5j–n). *Slthm27* mutant plants displayed significantly higher levels of *SlGAD2* transcripts and GABA contents under either normal or cold culture conditions (Fig. 5g; Fig. S12, see online supplementary material). These results suggested that silencing *SlTHM27* promotes GABA accumulation, reduces ROS levels, and improves cold tolerance in tomato seedlings. In conclusion, the SlTHM27 negatively regulates anthocyanin synthesis and tolerance to low temperature in tomato.

### SlTHM27 decreases *SlGAD2*-promoted cold tolerance in tomato by repressing *SlGAD2* transcription

**Figure 6:**
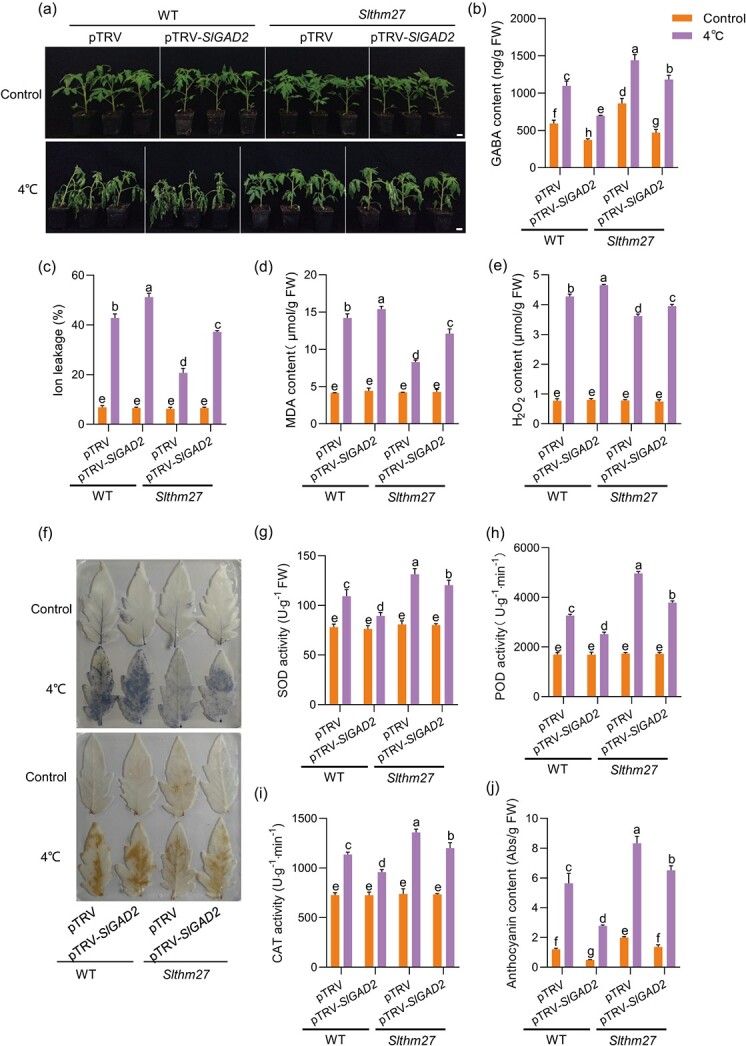
SlTHM27 negatively regulates cold tolerance in tomato by repressing *SlGAD2* transcription and GABA accumulation. (**a**) Phenotypic changes of silenced (pTRV-*SlGAD2*) or non-silenced *SlGAD2* (pTRV) in WT and *Slthm27* mutant plants before and after treatment at low temperature (4°C) for 4 days. Bar, 2.5 cm. (**b**) Endogenous GABA content tomato leaves shown in (**a**). (**c**–**e**) Electrolyte leakage (**c**), MDA content (**d**), and H_2_O_2_ content (**e**) of silenced (pTRV-*SlGAD2*) or non-silenced *SlGAD2* (pTRV) in WT and *Slthm27* mutant plants under control and cold stress (4°C). Plants were exposed to cold stress for 4 days, and leaves were collected for an ion leakage assay, MDA and H_2_O_2_ content measurement. (**f**) NBT and DAB staining of leaves of silenced (pTRV -*SlGAD2*) or non-silenced *SlGAD2* (pTRV) in WT and *Slthm27* mutant plants under control and cold stress (4°C). (**g**–**i**) SOD activity (**g**), POD activity (**h**), and CAT activity (**i**) of silenced (pTRV -*SlGAD2*) or non-silenced *SlGAD2* (pTRV) in WT and *Slthm27* mutant plants under control and cold stress. Plants were exposed to cold stress for 0 and 4 d and leaves were collected for the measurements. (**j**) Anthocyanin content of silenced (pTRV-*SlGAD2*) or non-silenced *SlGAD2* (pTRV) in WT and *Slthm27* mutant plants under control and cold stress (4°C). The different letters of the columns indicate significant differences (*P* < 0.05).

To further verify that SlTHM27 regulates tomato cold tolerance by regulating *SlGAD2*, we silenced *SlGAD2* in *Slthm27* mutant plants. The results indicated that under cold stress, there was a significant increase in the transcript level of *SlGAD2* in the *Slthm27* mutant compared to the WT with pTRV. In the background of the *Slthm27* mutant, where *SlGAD2* was silenced, the expression of *SlGAD2* was significantly suppressed, although it remained higher than the expression observed in the WT background with pTRV-*SlGAD2* (Fig. S13, see online supplementary material). We found that compared with pTRV in the WT background, the cold tolerance of pTRV in the *Slthm27* mutant background was significantly enhanced, with reduced ion leakage, MDA and H_2_O_2_ contents, and significantly increased antioxidant enzyme activities (SOD, POD, and CAT), along with significantly higher GABA and anthocyanin contents (Fig. 6a–j). However, in the *Slthm27* mutant background, knockdown of *SlGAD2* partially impaired cold tolerance in *Slthm27* mutant plants due to increased ion leakage level, MDA and H_2_O_2_ levels, and reduced antioxidant enzyme activity (Fig. 6a–i). Furthermore, under low temperature treatment, the GABA content of pTRV-*SlGAD2* in the *Slthm27* mutant background was more similar to that of pTRV in the WT background (Fig. 6b). It suggests that SlTHM27 regulates *SlGAD2* to affect GABA synthesis. Taken together, our results suggest that SlTHM27 negatively regulates cold tolerance in tomato by inhibiting *SlGAD2*-promoted GABA accumulation and anthocyanin biosynthesis.

## Discussion

### Exogenous spraying of 55 mM GABA effectively improved tomato cold tolerance

Tomato, originally from the tropics, is highly sensitive to low-temperature stress [33]. Therefore, elucidating the molecular mechanism of tomato cold sensitivity is important for crop breeding and improvement. GABA is not only a metabolic substance but also a signaling molecule that plays a critical role in mitigating cold injury in various species through accumulation [2, 36, 37]. In this study, tomato seedlings showed typical symptoms of cold injury under cold stress, including slow growth and reduction in fresh and dry weight, which were effectively alleviated by exogenous spraying of 55 mM GABA (Fig. 1). Moreover, we found that exogenous GABA maintained the integrity of cellular structure by improving the activity of antioxidant enzymes to scavenge ROS (Fig. 1). It was further demonstrated that exogenous GABA could regulate the activation capacity of plant antioxidant defense system under cold stress [37, 38]. Therefore, exogenous spraying of GABA is an effective way to improve the cold tolerance of tomato seedlings during tomato cultivation in facilities.

### *SlGAD2* is a positive regulator to improve the cold tolerance of tomato

GAD is a key enzyme in GABA production [28]. Our results showed that the tissue expression of the five *GAD* genes varied greatly, with all four *GAD* genes except *SlGAD5* being transcribed at higher levels in leaves (Fig. 2a–e); *SlGAD1* and *SlGAD2* being more highly expressed in ripe tomato fruits (Fig. 2a and b). Some studies have shown that the expression of *SlGAD1–3* was essential for the synthesis of GABA in tomato fruits [39]. The only difference was that the present study found lower expression of *SlGAD3* in ripe fruits, possibly due to the fact that the mRNA level of *SlGAD3* is highest early in fruit development and decreases with fruit ripening [40]. In addition, this study investigated the transcript levels of *SlGADs* in tomato leaves under cold stress for the first time (Fig. 2). Under cold stress, the transcript levels of *SlGAD1* and *SlGAD3* decreased and then increased, whereas the transcript level of *SlGAD4* showed a concurrent trend of increase and then decrease; only the relative expression of *SlGAD2* increased significantly with increasing stress time (Fig. 2f). It is noteworthy that the relative expression of *SlGAD2* was basically consistent with the dynamic changes of GABA content under cold stress. This implied that *SlGAD2* plays a crucial role in change of the GABA content of tomato seedlings under cold stress.

### *SlGAD2* positively regulates the cold tolerance in tomatoes by scavenging ROS and increasing anthocyanin content

We further investigated the mechanism of action of *SlGAD2*, given that *SlGAD2* was the most sensitive to cold stress. We found that overexpression of *SlGAD2* increased the cold tolerance of tomato seedlings by increasing GABA content and antioxidant capacity (Fig. S4, see online supplementary material). On the contrary, *Slgad2* mutants showed the opposite trend (Fig. 3). This was consistent with the conclusion that exogenous GABA improves cold tolerance by increasing antioxidant capacity in banana fruits [38]. However, it is not clear whether GABA improves cold resistance in tomato through other pathways. In this study, we found that anthocyanin content was significantly accumulated in *SlGAD2*-overexpressing plants (Fig. S5, see online supplementary material). Anthocyanins are a major class of flavonoids whose synthesis and accumulation are induced by low temperature [17, 18]. The anthocyanin content and the expression of anthocyanin-related genes were significantly increased in *SlGAD2* overexpressing plants under low-temperature stress, whereas the anthocyanin content was significantly suppressed in *Slgad2* mutant plants (Fig. 3j–n). Anthocyanins can effectively scavenge excessive ROS to maintain normal cellular redox homeostasis under abiotic stress [10]. Therefore, anthocyanins accumulated in the overexpressed *SlGAD2* transgenic tomato attenuated low temperature induced oxidative damage. At the same time, we found that spraying 55 mM GABA increased the content of endogenous GABA, which also promoted the anthocyanin accumulation and improved cold tolerance in tomato (Fig. S6, see online supplementary material). These results further demonstrated that GABA accumulation could efficiently scavenge ROS through both enzymatic and non-enzymatic antioxidant systems under cold stress, and *SlGAD2* played an essential role in this process. In addition, it will be interesting to study how GABA triggers the expression of anthocyanin synthesis genes.

### SlTHM27 negatively regulated cold by inhibiting *SlGAD2* transcription

Most of the MYB class TFs can respond positively to plant tolerance to abiotic stress [41], only a few MYB class TFs were negative regulators of abiotic stress response [42, 43]. For example, VcMYB4a, which has an EAR repressor domain of structure, was down-regulated by low-temperature treatment, and blueberry healing tissues exhibited a cold-sensitive phenotype after *VcMYB4a* overexpression [44]. Similarly, we found that the SlTHM27 protein sequence contained a C-terminal EAR repressor motif (Fig. S10, see online supplementary material), and its mutant plants exhibited enhanced cold tolerance (Fig. 5). In addition, MYB class transcriptional activators and repressors have been widely explored in the regulation of anthocyanin biosynthesis [35, 45]. AtMYB4, which contains an EAR motif, represses C4H expression [46]. MdMYB16 inhibits anthocyanin synthesis in apple healing tissues by repressing *MdANS* and *MdUFGT* expression [35]. In this study, we found that SlTHM27 is an MdMYB16 homolog with 66.67% protein sequence similarity. SlTHM27 was found to repress *SlGAD2* transcription by qRT-PCR and Dual-LUC assays (Fig. 4e; Fig. S12, see online supplementary material). Furthermore, our results convincingly demonstrated that *SlGAD2* acts downstream of SlTHM27 (Figs 4 and 6). Thus, the enhanced cold tolerance of *Slthm27* mutant plants depends on the increased transcript level of *SlGAD2*, which in turn promotes GABA accumulation and improves anthocyanin content and ROS scavenging (Figs 5 and 6).

**Figure 7:**
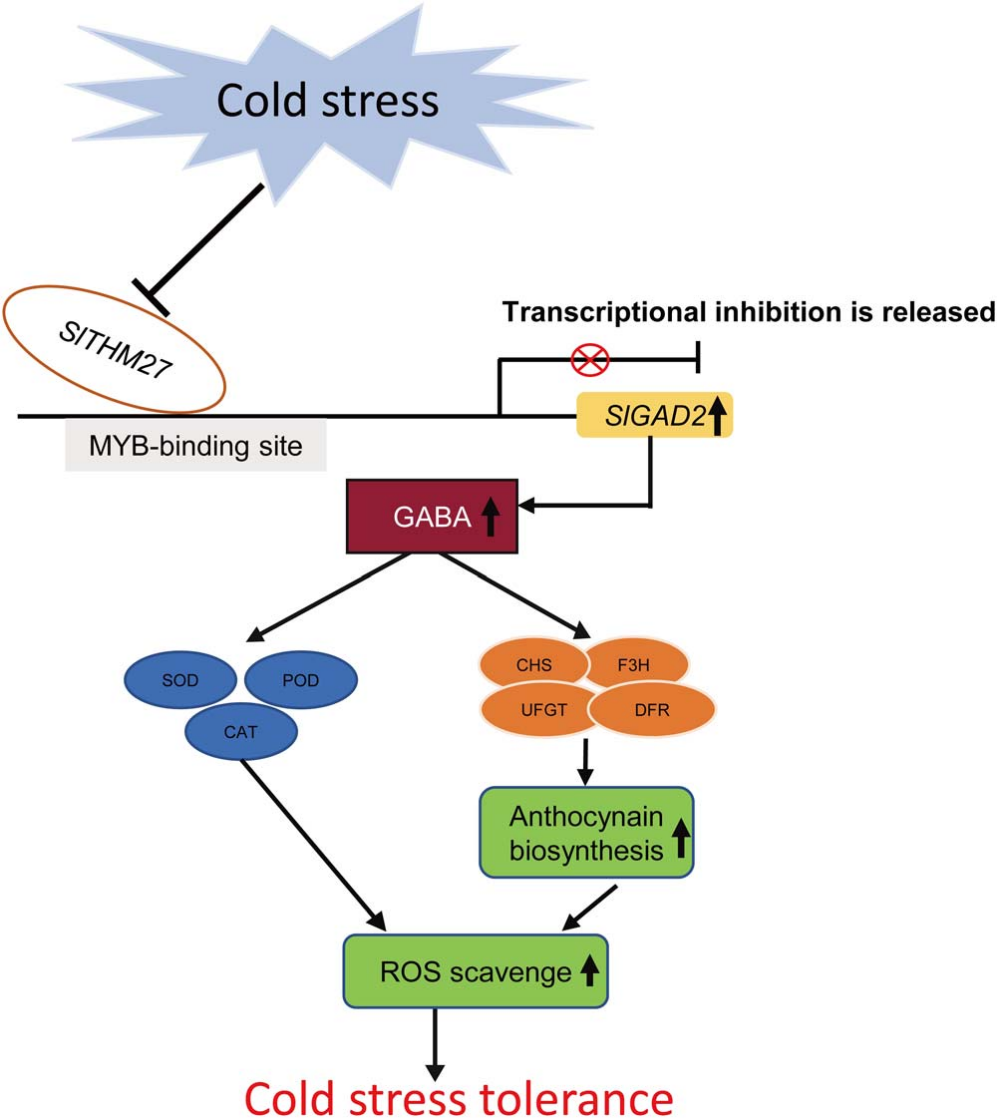
A working model for SlTHM27-*SlGAD2* in response to cold stress. Under low-temperature stress, the transcript level of *SlTHM27* was repressed, thereby increasing the expression level of *SlGAD2*. *SlGAD2* promoted the accumulation of GABA, which increased antioxidant enzyme activities and anthocyanin levels. This helped to scavenge excess reactive oxygen species (ROS) and improved cold tolerance in tomato.

Taken together, our work has revealed for the first time the mechanism of the SlTHM27-*SlGAD2* regulatory module responds to cold stress by regulating GABA levels (Fig. 7). Cold stress inhibited the mRNA level of the negative regulator *SlTHM27* to weaken the transcriptional repression of *SlGAD2* and induced the synthesis of GABA to improve tomato resistance through enzymatic and non-enzymatic antioxidant systems. Our study provides valuable insights for improving cold tolerance in tomato.

## Materials and methods

### Vector construction and genetic transformation

The CDS amplicon of *SlGAD2* gene (ID: Solyc11g011920.1) was inserted into pHellsgate2 vector, and the fusion-expressing vector was introduced into the tomato ‘Ailsa Craig’ (WT) to achieve genetic transformation. The *SlGAD2* overexpression plants were produced and identified by PCR. The primers used in this research are listed in Table S1 (see online supplementary material).

To edit the *SlTHM27 and SlGAD2* genes using the CRISPR/Cas9 system. Targets were designed and selected using Cas-Designer (http://www.rgenome.net/cas-designer/). The guide RNA sequence was constructed and inserted into pBSE402 [47]. Tomato transformation was performed according to previously described methods. Positively transformed plants were identified by extracting genomic DNA from stable transgenic lines, cloning potential editing fragments of *SlTHM27 and SlGAD2*, respectively, and sequencing them.

For gene silencing, primers listed in Table S2 (see online supplementary material) were used to amplify cDNA fragment of *SlGAD2*, and the PCR amplification product were then transformed into the TRV2 vector. The fusion expression vector was transfected into wild-type tomato cotyledons to generate *SlGAD2* gene-silencing plants. For details of the method, please refer to our group’s previous study [48].

### Plant materials and treatment

We germinated both WT and transgenic tomato seeds at 28°C, after which they were planted separately in cavity trays and cultured in a light incubator under culture conditions: 25°C/16°C (day/night). Seedlings were transplanted to nurseries with four fully expanded true leaves, and continued to be grown until the fifth true leaf was fully expanded under environmental conditions as described previously. For the cold treatment, tomato seedlings were incubated in a cold chamber at 4°C for 4 days.

In the exogenous GABA treatment assays, leaves were uniformly sprayed with distilled water (0 mM GABA) or GABA solutions of appropriate concentrations, 10 mL plant^−1^. Leaves were treated for 12 h at 25°C and then subjected to a low temperature treatment (4°C). The GABA concentrations applied included C0 (0 mM GABA), C40 (40 mM GABA), C55 (55 mM GABA), C60 (60 mM GABA), and C70 (70 mM GABA).

### RT-qPCR analysis

Total RNA was extracted with tomato leaves, and reverse transcription did with PrimeScriptTM RT Kit (Takara Bio, Shiga, Japan). RT-qPCR analysis was performed with ChamQ SYBR qPCR Master Mix (Vazyme, Nanjing, China). *SlACTIN* was employed as the parameter.

### Bioinformatics analysis

DNAMAN was used for multiple amino acid comparisons of protein sequences, and the SMART program was used to analyse conserved protein structural domains [49]. The online tool PlantTFDB (http://planttfdb.gao-lab.org/prediction.php) was used to analyse the TF binding sites in the *SlGAD2* promoter.

### SlTHM27-GFP subcellular localization

The CDS sequences of *SlTHM27* was inserted into pAC402-GFP. The fusion proteins were transferred to one-month-old tobacco leaves. GFP signal was detected by laser scanning confocal microscopy (TCS-SP8 SR; Leica, Wetzlar, Germany). NLS-mCherry was used to as the nucleus marker.

### Yeast one-hybrid (Y1H) assay

The *SlGAD2* promoter was introduced into the pAbAi vector and then digested with BbsI (NEB, Ipswich, MA, USA) as a bait. The CDS sequences of *SlTHM27* was cloned into pGADT7 as a prey vector. Y1H experiments were performed according to the Matchmaker Gold Y1H manufacturer’s instructions. Table S1 (see online supplementary material) lists the primers used for amplification.

### Dual-luciferase reporter assay system

For the LUC assay, the fusion reporter gene (*proSlGAD2*) and effector (*SlTHM27*) plasmids were inserted into *Agrobacterium tumefaciens* GV3101, respectively. One-month-old tobacco leaves were transiently transformed as described previously [50]. A dual luciferase reporter assay system (Promega) was used to detect luciferase activity. In low temperature treatment, tobacco plants were subjected to treatment at 4°C for 3 h and then proteins were extracted. Ten independent biological samples were used.

### EMSA

The truncated *SlTHM27* was cloned into pMAL-c5X. The resulting plasmid was converted into *Escherichia coli* strain BL21 (DE3) and amplified for 8 h at 28°C. MBP-SlTHM27 fusion protein was purified using straight-chain starch resin (NEB, E8201S, USA). The EMSA assays were carried out with the Light Shift Chemiluminescent EMSA Kit (ThermoFisher Scientific). Table S1 (see online supplementary material) lists the primers used for amplification.

### Determination of GABA content

GABA was extracted using 0.1 g of tomato leaves, and GABA content was measured by LC–MS [51]. GABA content was analysed using three independent experiments each having three replicates.

### Determination of total anthocyanin content

Total anthocyanins were extracted from tomato leaves using a solution of methanol and HCl (0.1%, v/v) at 4°C overnight. Absorbance was detected at 530, 657 nm using a UV–Vis spectrophotometer (Shimadzu UV-1780) [10].

### H_2_O_2_, ion leakage, enzymes activities, and MDA content measurements

H_2_O_2_ were determined as previously described by Xie *et al.* [50]. Ion leakage was measured according to the method of Jiang *et al.* [52]. SOD, POD, CAT activities, and MDA levels were calculated as previously described [29].

### DAB and NBT staining of the tomato leaves

Tomato leaves were analysed for H_2_O_2_ by placing them in a 1 mg mL^−1^ solution of 3,3′-diaminobenzidine (DAB) (pH 3.8) in the light for 8 h. For O_2_^−^ analysis, the tomato leaves were immersed in a 0.5 mg mL^−1^ solution of nitroblue tetrazolium (NBT) in the dark for 8 h.

### Determination of growth

The height of tomato seedlings was measured in cm using a meterstick and stem thickness in mm using a Vernier caliper. Fresh and dry weights of tomato seedlings were measured using a balance with accuracy to one thousandth of a millimeter.

### Statistical analysis

Data are presented as mean ± SD. Statistical analysis was performed with SPSS 23.0. One-way ANOVA was used to consider differences significant at *P* < 0.05 or *P* < 0.01 (Tukey’s test).

## Acknowledgements

This work was supported by Scientific and Technological Innovative Research Team of Shaanxi Province (2021TD-34), China Agriculture Research System (CARS-23-D06), and the National College Student Innovation and Entrepreneurship Training Program (202210712083 and X202310712703). The authors thank the Horticulture Science Research Center at College of Horticulture, Northwest A&F University for their technical support in this work. The authors are grateful to Jing Zhang (Horticulture Science Research Center, Northwest A&F University, Yangling, China) for providing professional technical assistance with LC–MS analysis.

## Author contributions

X.H., T.L., J.W., and Y.Z. conceived and designed the experiments. J.W. and X.H. wrote the paper. J.W. and Y.Z. performed the experiments. J.W., A.K., Z.K., Y.M., J.Z., and H.D. provided advice related to the research. All authors read and approved the manuscript for submission.

## Data availability

All relevant data for this study are provided in this article and its supplements.

## Conflict of interest statement

The authors declare no competing interests.

## Supplementary data

Supplementary data is available at *Horticulture Research* online.

## Supplementary Material

Web_Material_uhae096
