# Supplementary material for: SlGAD2 is the target of SlTHM27, positively regulates cold tolerance by mediating anthocyanin biosynthesis in tomato
Source: Hortic Res. 2024 Apr 4;11(6):uhae096. doi: 10.1093/hr/uhae096 (PMC11161262; doi:10.1093/hr/uhae096)
Supplement: Web_Material_uhae096 [file web_material_uhae096.zip › Fig.S2.pdf]

```
SlGAD1     .MVLSKTP..SDDSVHSTFASRYVRTSLPRFEMLEKSIPKEAAYQMINDELMLDGNPRLNLASFVT   63
SlGAD2     MVLTTTSIRDSEESLHCTFASRYVQEPLPKFKIPKKSMPKEAAYQIVNDELMLDGNPRLNLASFVS   66
SlGAD3     .MVLSKTSSESDVSVHSTFASRYVRTSLPRFEMPENSIPKEAAYQIINDELMLDGNPRLNLASFVT   65
SlGAD4     .MVISKAASESDLSVHSTFASRYVRVSLPRFKMPENSIPKDAAYQIINDELMLDGNPRLNLASFVT   65
SlGAD5     .MVLSKIASESDVSVHSTFASRYVRTSLPRFKMPENSIPKEAAYQIINDELMLDGNPRLNLASFVT   65
Consensus             s s h tfasryv  lp f   s pk aayq ndelmldgnprlnlasfv

SlGAD1     TWMEPECDKLMMASINKNYVDMDEYPVTTELQNRCVNMIARLFNAPLKEEEIGIGVGTVGSSEAIM  129
SlGAD2     TWMEPECDKLIMSSINKNYVDMDEYPVTTELQNRCVNMLAHLFHAPVGDDETAVGVGTVGSSEAIM  132
SlGAD3     TWMEPECDKLMMSSINKNYVDMDEYPVTTELQNRCVNMIARLFNAPLEEEEAAIGVGTVGSSEAIM  131
SlGAD4     TWMEPECDKLIMDSINKNYVDMDEYPVTTELQNRCVNMIAHLFNAPLGEGEAAVGVGTVGSSEAIM  131
SlGAD5     TWMEPECDKLMMDSINKNYVDMDEYPVTTELQNRCVNMIAHLFNAPLEDGETAVGVGTVGSSEAIM  131
Consensus  twmepecdkl m sinknyvdmdeypvttelqnrcvnm a lf ap  e  gvgtvgsseaim

SlGAD1     LAGLAFKRNWQNKRKAEGKPYDKPNIVTGANVQVCWEKFANYFEVELKQVKLSEGYYVMDPIKAVE  195
SlGAD2     LAGLAFKRKWQSKRKAEGKPFDKPNIVTGANVQVCWEKFARYFEVELKEVKLKEGYYVMDPAKAVE  198
SlGAD3     LAGLAFKRNWQNKRKAEGKPYDKPNIVTGANVQVCWEKFANYFEVELKEVKLREGYYVMDPMKAVE  197
SlGAD4     LAGLAFKRKWQNKMKAQGKPHDKPNIVTGANVQVCWEKFARYFEVELKEVKLEDGYYVMDPEKAVE  197
SlGAD5     LAGLAFKRKWQNKMKAQGKPYDKPNIVTGANVQVCWEKFARYFEVELKEVKLTDGYYVMSPEKAVE  197
Consensus  laglafkr wq k ka gkp dkpnivtganvqvcwekfa yfevelk vkl gyyvm p kave

SlGAD1     MVDDNTICVAAILGSTLNGEFEDVKLLNDLLIEKNKQTGWDTPIHVDAASGGFIAPFIYPELEWDF  261
SlGAD2     IVDENTICVAAILGSTLTGEFEDVKLLNELLTKKNKETGWETPIHVDAASGGFIAPFLWPDLEWDF  264
SlGAD3     MVDDNTICVAAILGSTLNGEFEDVKLLNDLLIQKNKQTGWDTPIHVDAASGGFIAPFIYPELEWDF  263
SlGAD4     MVDENTICVAAILGSTLNGEFEDVKRLNDLLVEKNKETGWDTPIHVDAASGGFIAPFIYPEIEWDF  263
SlGAD5     MVDENTICVAAILGSTLNGEFEDVKKLNDLLIEKNKETGWDTPIHVDAASGGFIAPFIYPELEWDF  263
Consensus   vd nticvaailgstl gefedvk ln ll  knk tgw tpihvdaasggfiapf  p ewdf

SlGAD1     RLPLVKSINVSGHKYGLVYAGIGWVIWRTKQDLPQQLIFHINYLGADQPTFTLNFSKGSSQVIAQY  327
SlGAD2     RLPLVKSINVSGHKYGLVYAGVGWVIWRSKEDLPDELVFHINYLGSDQPTFTLNFSKGSYQIIAQY  330
SlGAD3     RLPLVKSINVSGHKYGLVYAGIGWVIWRTKQDLPEQLIFHINYLGADQLTFTLNFSKGSSQIIAQY  329
SlGAD4     RLPLVKSINVSGHKYGLVYAGIGWVIWRNKEDLPEELIFHINYLGADQPTFTLNFSKGSSQVIAQY  329
SlGAD5     RLPLVKSINVSGHKYGLVYAGIGWVIWRNKEDLPDELIFHINYLGADQPTFTLNFSKGSSQVIAQY  329
Consensus  rlplvksinvsghkyglvyag gwviwr k dlp l fhinylg dq tftlnfskgs q iaqy

SlGAD1     YQLIRLGYEGYRNVMENCRENAIVLRKGLEKTGRFNIISKDEGIPLVAFSLKDNSLHNEFEVSETL  393
SlGAD2     YQLIRLGFEGYKDVMKNCLSNAKVLTEGITKMGRFDIVSKDVGVPVVAFSLRDSSKYTVFEVSEHL  396
SlGAD3     YQLIRLGYEGYRNVMENCRENAIVLREGLEKTGRFNIVSKDEGVPLVAFSLKDNSLHNEFEVSETL  395
SlGAD4     YQLIRLGYEGYKNIMENCQENTRVLREGLEKIERFNIVSKEIGVPLVAFSLKDNSKHNEFEISEHL  395
SlGAD5     YQLIRLGYEGYKNVMENCQENARVLREGLEKTGRFEIVSKEVGVPLVAFSLKDNSKHDEFEISETL  395
Consensus  yqlirlg egy  m nc n vl g k rf i sk g p vafsl d s  fe se l

SlGAD1     RRFGWIVPAYTMPADLQHVTVLRVVIREDFSRTLADRLVSDIVKVLHELPNAK.......KVEDNL  452
SlGAD2     RRFGWIVPAYTMPPDAEHIAVLRVVIREDFSHSLAERLVSDIEKILSELDTQPPRLPTKAVRVTAE  462
SlGAD3     RRFGWIVPAYTMPADAQHITVLRVVIREDFSRTLAERLVFDIVKVLHELDTLPARL..SAKMEENL  459
SlGAD4     RRFGWIVPAYTMPANAEHVTVLRVVIREDFSRTLAERLVGDIVKVLHELDNLPARV..TAKLAAI.  458
SlGAD5     RRFGWIVPAYTMPPDAQHITVLRVVIREDFSRTLAERLVMDIVKVLHELDMLPARV..KAKLAVAE  459
Consensus  rrfgwivpaytmp   h vlrvviredfs la rlv di k l el

SlGAD1     MINNE..K......KTEIEVQRAIAEFWKKYVLARKASIC...                        484
SlGAD2     EVRDDKGDGLHHFHMDTVETQKDIIKHWRKIAGK....KTSGV                        501
SlGAD3     VIENNNGK......KTEIEVQREVTDFWKKFVLARKAAVC...                        493
SlGAD4     ....AEGEGSSGHKKSPMEVQLEITNVWKKFVAEKKKTKNVIC                        497
SlGAD5     EAAAANGIDSVAHHKTDREWELQVTEAWKKFVADKKKNKTMGV                        502
Consensus                     e         w k
```
