# Supplementary material for: SlGAD2 is the target of SlTHM27, positively regulates cold tolerance by mediating anthocyanin biosynthesis in tomato
Source: Hortic Res. 2024 Apr 4;11(6):uhae096. doi: 10.1093/hr/uhae096 (PMC11161262; doi:10.1093/hr/uhae096)
Supplement: Web_Material_uhae096 [file web_material_uhae096.zip › Fig.S7.pdf]

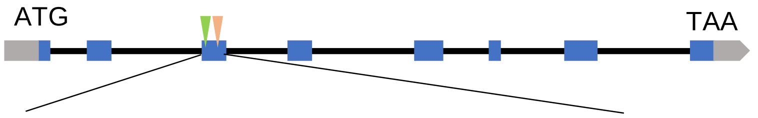

sg1: WT 5'-TTAATGCACCACTTGAAGA-3'

Slgad2 #2 5'-TTAATGCACCA ----AAGA-3'

Slgad2 #3 5'-TTAATGCACCACTTGAAAGA-3'

sg2: WT 5'-AGGAAAGCCCTATGATAAG-3'

Slgad2 #2 5'-AGG ------CCTATGATAAG-3'

Slgad2 #3 5'-AGGA--GC-CTATGATAAG-3'
