# Supplementary material for: SlGAD2 is the target of SlTHM27, positively regulates cold tolerance by mediating anthocyanin biosynthesis in tomato
Source: Hortic Res. 2024 Apr 4;11(6):uhae096. doi: 10.1093/hr/uhae096 (PMC11161262; doi:10.1093/hr/uhae096)
Supplement: Web_Material_uhae096 [file web_material_uhae096.zip › Fig.S8.pdf]

Identity=66.67%

```
MdMYB16     MGRSPCCEKAHTNKGAWTKEEDDRLIAYIRAHGEGCWRSLPKAAGLLRCGKSCRLRWINYLRPDLK   66
SlTHM27     MGRSPCCEKAHTNKGAWTKEEDERLISYIRAHGEGCWRSLPKAAGLLRCGKSCRLRWINYLRPDLK   66
Consensus   mgrspccekahtnkgawtkeed rli yirahgegcwrslpkaagllrcgkscrlrwinylrpdlk

MdMYB16     RGNFTEEEDELIIKLHSLLGNKWSLIAGRLPGRTDNEIKNYWNTHIRRKLLTRGIDPTTHRPLNE.  131
SlTHM27     RGNFTEEEDELIIKLHSLLGNKWSLIAGRLPGRTDNEIKNYWNTHIRRKLLSRGIDPTTHRSINDP  132
Consensus   rgnfteeedeliiklhsllgnkwsliagrlpgrtdneiknywnthirrkll rgidptthr  n

MdMYB16     TPQESATTISFAAASANIKEE...DKKISITNGLVCKDSKNPVQERC........PDLNLDLQISP  186
SlTHM27     TTIPKVTTITFAAAHENIKDIDQQDEMINIKAEFVETSKESDNNEIIQEKSSSCLPDLNLELRISP  198
Consensus   t     tti faaa  nik     d  i i    v         e          pdlnl l isp

MdMYB16     PCQPQQPSDGLKSGGRGLCFSCSLGLQDAKNCSCGRDAIGGAT.....SGTTNIGYDFLGLKNGV.  246
SlTHM27     PHHQQLDHHRHHQRSSSLCFTCSLGIQNSKDCSCGSESNGNGWSNNMVSMNIMAGYDFLGLKTNGL  264
Consensus   p   q            lcf csl g q  k cscg    g        s     gydflglk

MdMYB16     LDYRSLEM                                                            254
SlTHM27     LDYRTLET                                                            272
Consensus   ldyr le
```
