# Supplementary material for: SlGAD2 is the target of SlTHM27, positively regulates cold tolerance by mediating anthocyanin biosynthesis in tomato
Source: Hortic Res. 2024 Apr 4;11(6):uhae096. doi: 10.1093/hr/uhae096 (PMC11161262; doi:10.1093/hr/uhae096)
Supplement: Web_Material_uhae096 [file web_material_uhae096.zip › Fig.S9.pdf]

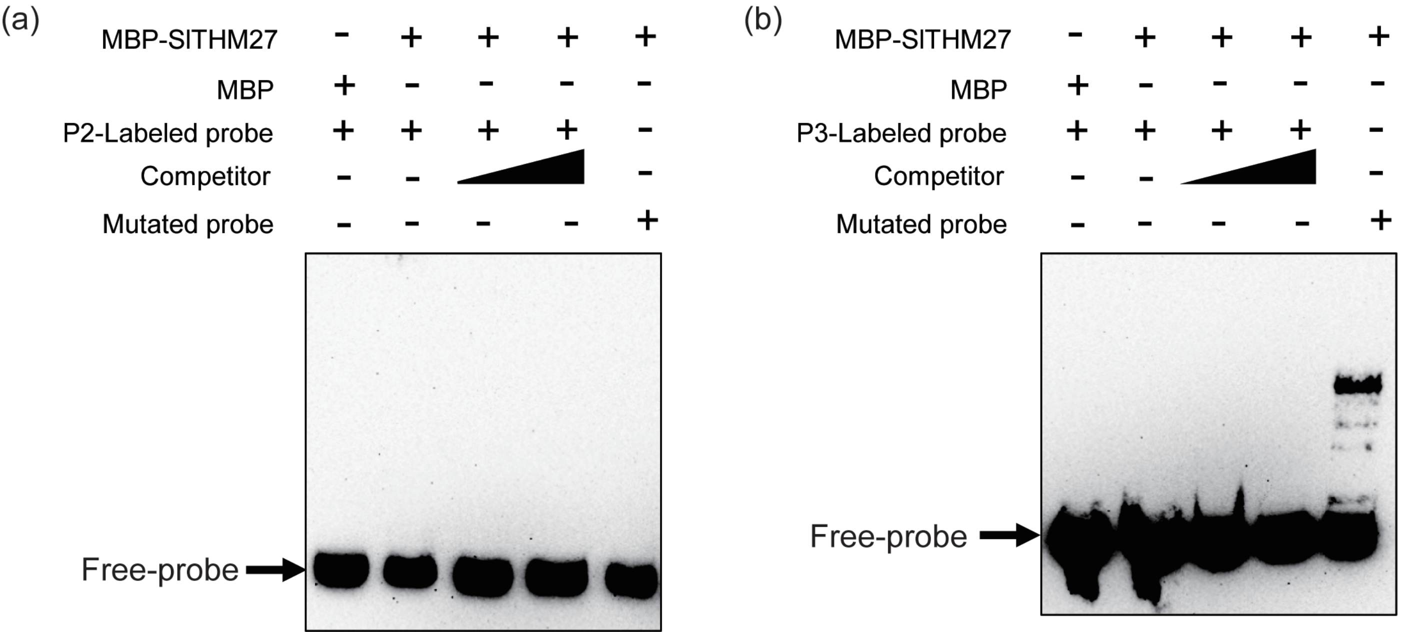

(a)
MBP-SlTHM27
MBP
P2-Labeled probe
Competitor
Mutated probe
Free-probe
(b)
MBP-SlTHM27
MBP
P3-Labeled probe
Competitor
Mutated probe
Free-probe
