# Supplementary material for: SlGAD2 is the target of SlTHM27, positively regulates cold tolerance by mediating anthocyanin biosynthesis in tomato
Source: Hortic Res. 2024 Apr 4;11(6):uhae096. doi: 10.1093/hr/uhae096 (PMC11161262; doi:10.1093/hr/uhae096)
Supplement: Web_Material_uhae096 [file web_material_uhae096.zip › Fig.S10.pdf]

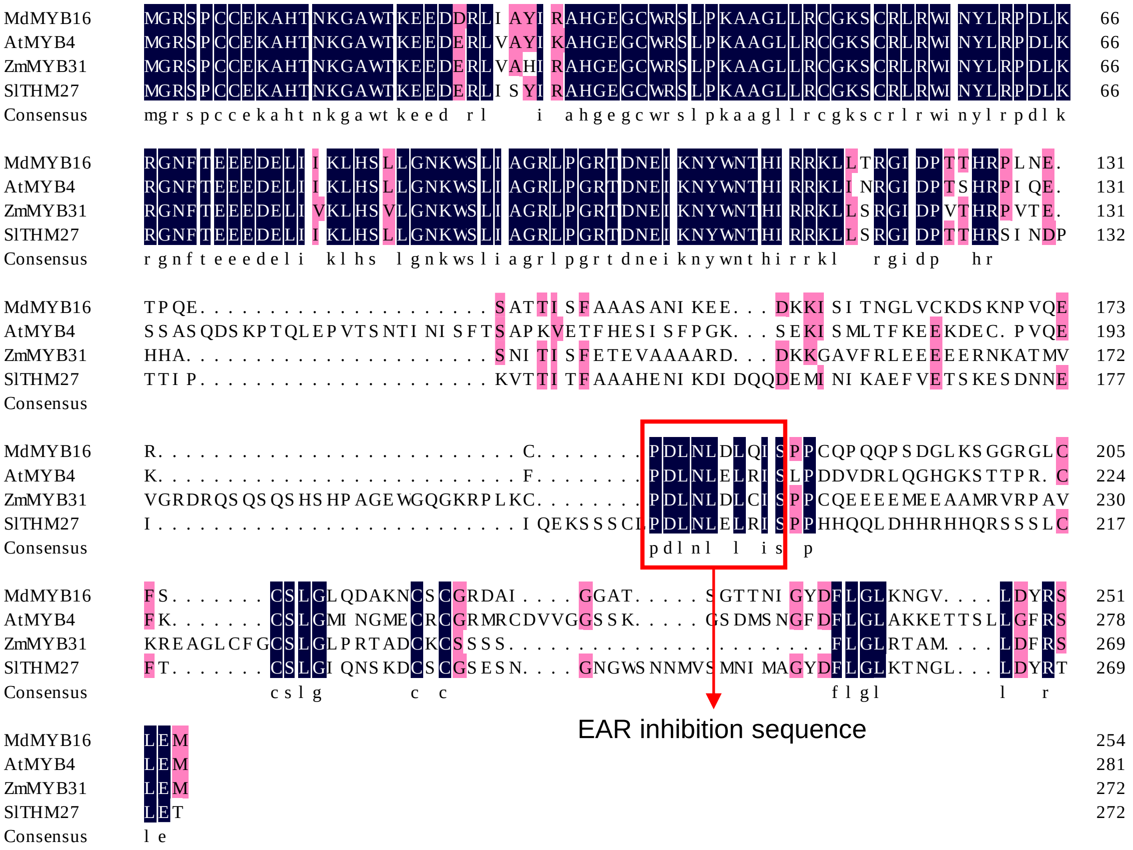
MdMYB16 MGRSPCCEKAHTNKGAWTKEEDDRLIAYIRAHGEGCWRSLPKAAGLLRCGKSCRLRWINYLRPDLK 66
AtMYB4 MGRSPCCEKAHTNKGAWTKEEDERLVAYIKAHGEGCWRSLPKAAGLLRCGKSCRLRWINYLRPDLK 66
ZmMYB31 MGRSPCCEKAHTNKGAWTKEEDERLVAHIRAHGEGCWRSLPKAAGLLRCGKSCRLRWINYLRPDLK 66
SlTHM27 MGRSPCCEKAHTNKGAWTKEEDERLISYIRAHGEGCWRSLPKAAGLLRCGKSCRLRWINYLRPDLK 66
Consensus mgrspccekahtnkgawtkeed rl i ahgegcwrslpkaagllrcgkscrlrwinylrpdlk
MdMYB16 RGNFTEEEDELIIKLHSLLGNKWSLIAGRLPGRTDNEIKNYWNTHIRRKLLTRGIDPTTHRPLNE. 131
AtMYB4 RGNFTEEEDELIIKLHSLLGNKWSLIAGRLPGRTDNEIKNYWNTHIRRKLINRGIDPTSHRPIQE. 131
ZmMYB31 RGNFTEEEDELIVKLHSVLGNKWSLIAGRLPGRTDNEIKNYWNTHIRRKLLSRGIDPVTHRPVTE. 131
SlTHM27 RGNFTEEEDELIIKLHSLLGNKWSLIAGRLPGRTDNEIKNYWNTHIRRKLLSRGIDPTTHRSINDP 132
Consensus rgnfteeedeli klhs lgnkwsliagrlpgrtdneiknywnthirrkl rgidp hr
MdMYB16 TPQE.....................SATTISFAAASANIKEE...DKKISITNGLVCKDSKNPVQE 173
AtMYB4 SSASQDSKPTQLEPVTSNTINISFTSAPKVETFHESISFPGK...SEKISMLTFKEEKDEC.PVQE 193
ZmMYB31 HHA......................SNITISFETEVAAAARD...DKKGAVFRLEEEEERNKATMV 172
SlTHM27 TTIP.....................KVTTITFAAAHENIKDIDQQDEMINIKAEFVETSKESDNNE 177
Consensus
MdMYB16 R..........................C........PDLNLDLQISPPCQPQQPSDGLKSGGRGLC 205
AtMYB4 K..........................F........PDLNLELRISLPDDVDRLQGHGKSTTPR.C 224
ZmMYB31 VGRDRQSQSQSHSHPAGEWGQGKRPLKC........PDLNLDLCISPPCQEEEEMEEAAMRVRPAV 230
SlTHM27 I..........................IQEKSSSCLPDLNLELRISPPHHQQLDHHRHHQRSSSLC 217
Consensus pdlnl l is p
MdMYB16 FS.......CSLGLQDAKNCSCGRDAI....GGAT.....SGTTNIGYDFLGLKNGV....LDYRS 251
AtMYB4 FK.......CSLGMINGMECRCGRMRCDVVGGSSK.....GSDMSNGFDFLGLAKKETTSLLGFRS 278
ZmMYB31 KREAGLCFGCSLGLPRTADCKCSSSS.......................FLGLRTAM....LDFRS 269
SlTHM27 FT.......CSLGIQNSKDCSCGSESN....GNGWSNNMVSMNIMAGYDFLGLKTNGL...LDYRT 269
Consensus cslg c c flgl l r
EAR inhibition sequence
MdMYB16 LEM 254
AtMYB4 LEM 281
ZmMYB31 LEM 272
SlTHM27 LET 272
Consensus le
