# Supplementary figures and images for: SlGAD2 is the target of SlTHM27, positively regulates cold tolerance by mediating anthocyanin biosynthesis in tomato
Source: Hortic Res. 2024 Apr 4;11(6):uhae096. doi: 10.1093/hr/uhae096 (PMC11161262; doi:10.1093/hr/uhae096)

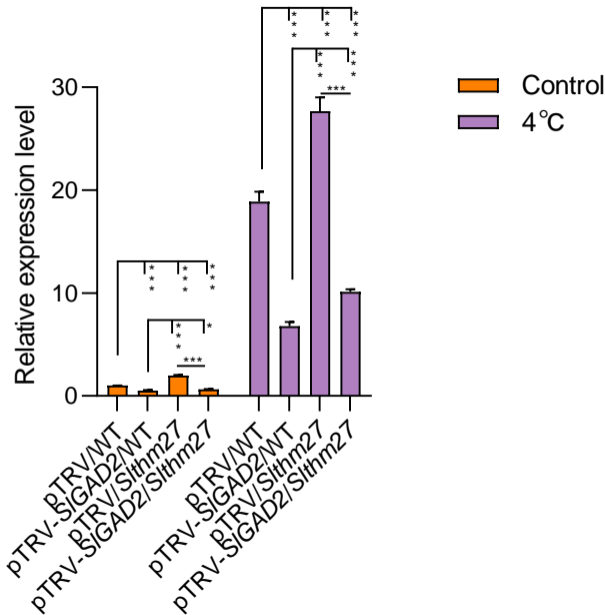

Supplement: Web_Material_uhae096 [file web_material_uhae096.zip › Fig. S13.pdf]

## GABA

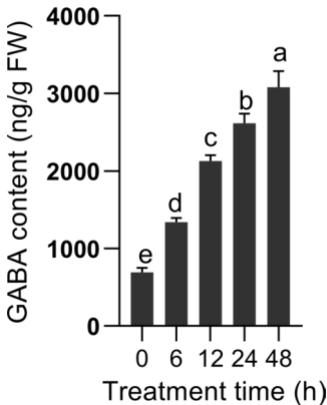

Supplement: Web_Material_uhae096 [file web_material_uhae096.zip › Fig.S1.pdf]

# *S/GAD2*

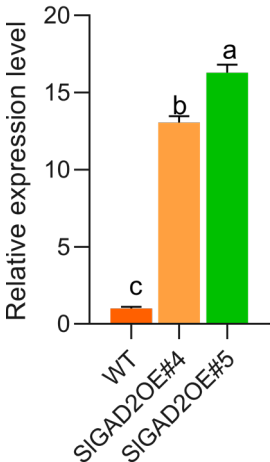

Supplement: Web_Material_uhae096 [file web_material_uhae096.zip › Fig.S3.pdf]

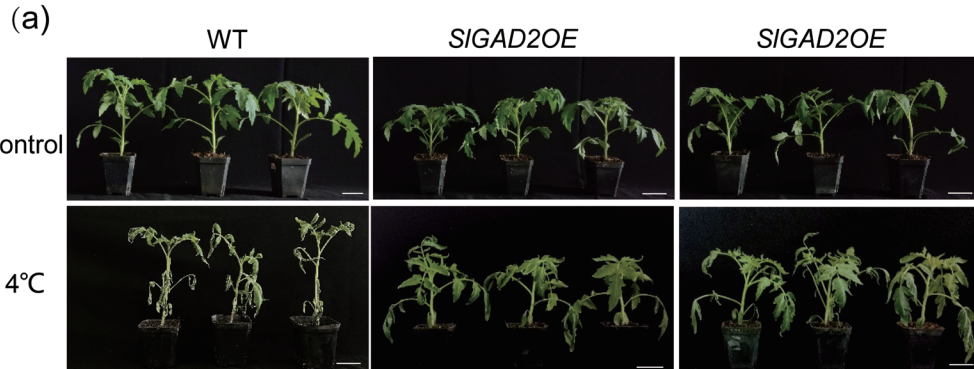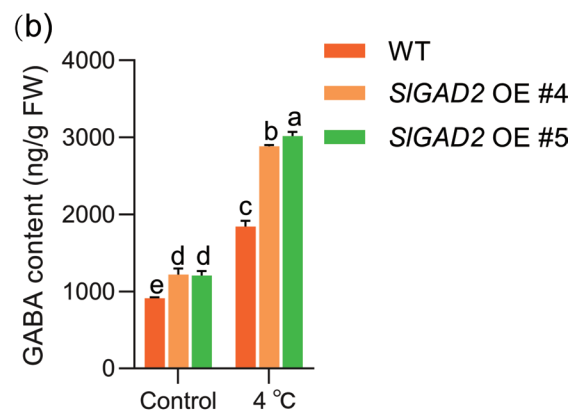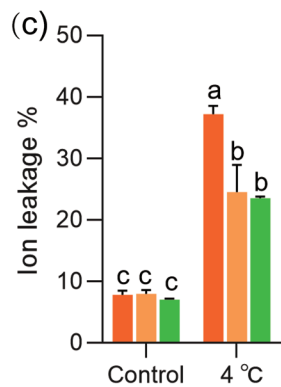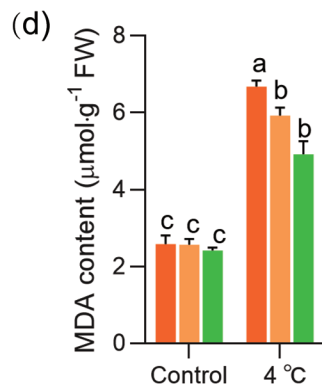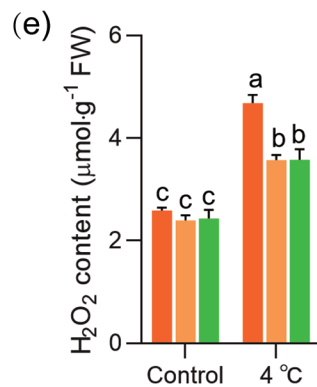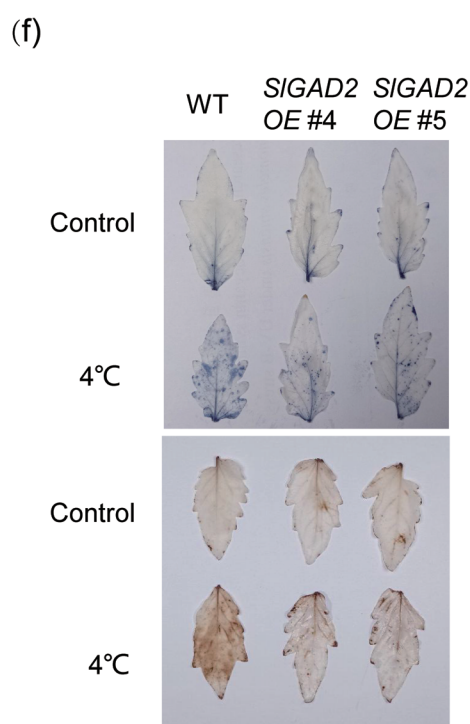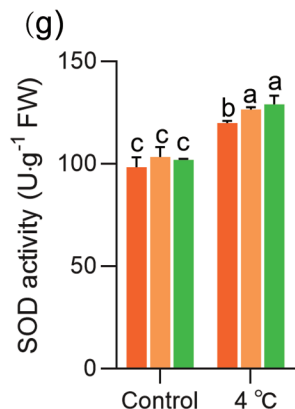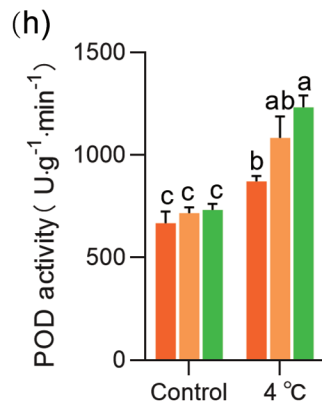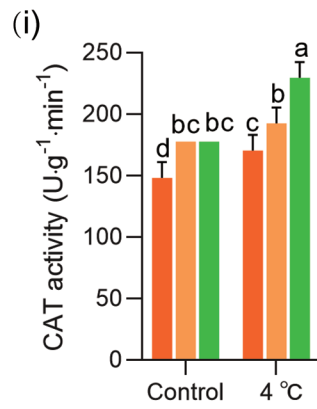

Supplement: Web_Material_uhae096 [file web_material_uhae096.zip › Fig.S4.pdf]

(a)

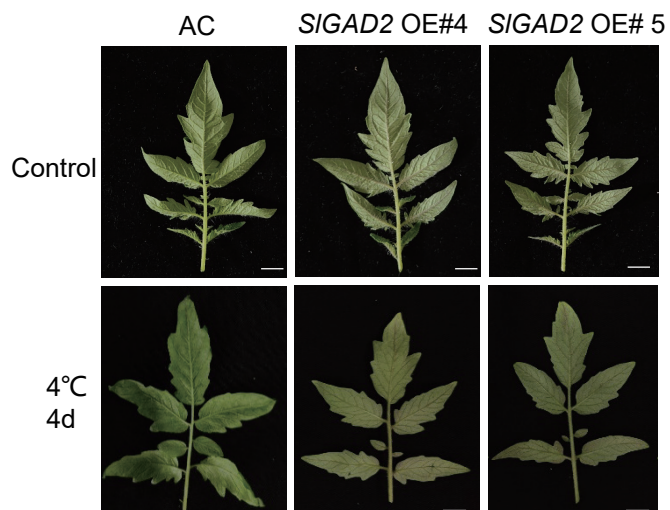

(b)

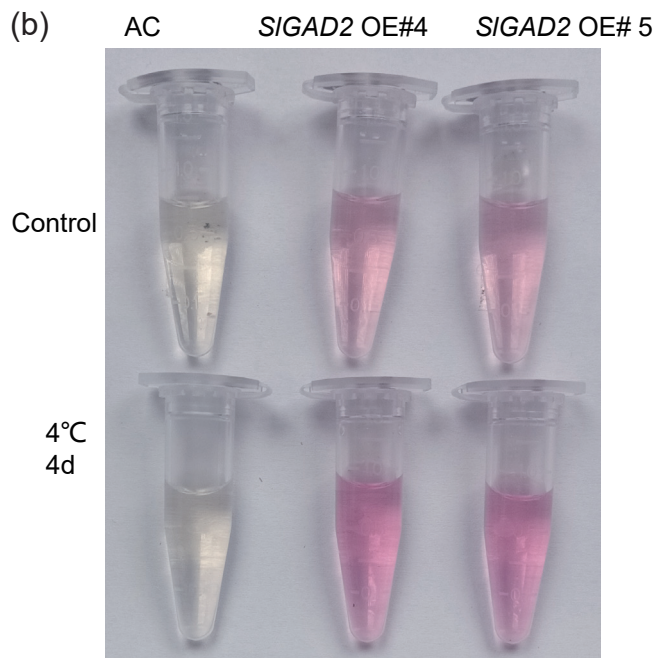

(c)

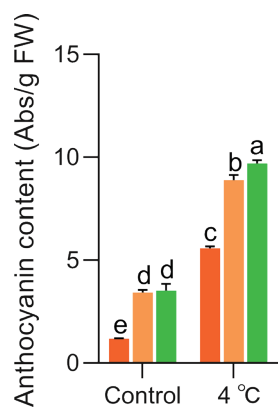

(d)

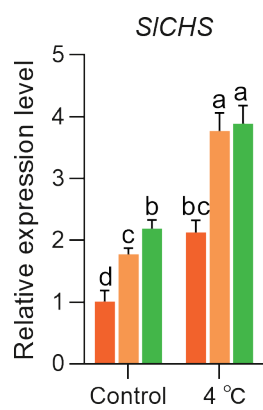

(e)

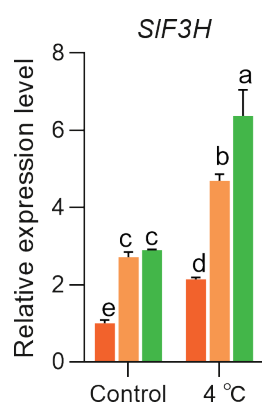

(f)

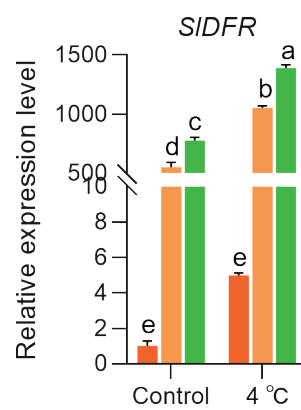

(g)

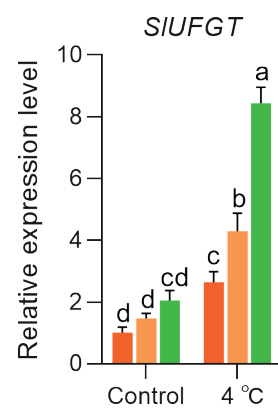

WT SIGAD2 OE#4 SIGAD2 OE#5

Supplement: Web_Material_uhae096 [file web_material_uhae096.zip › Fig.S5.pdf]

(a)

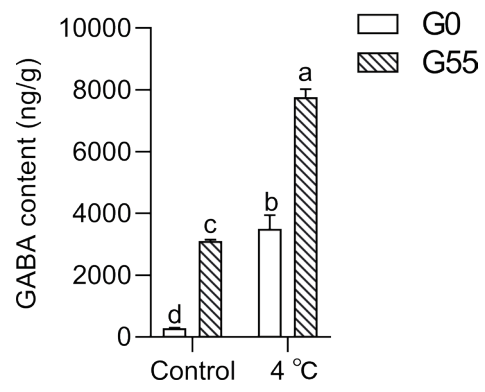

(b)

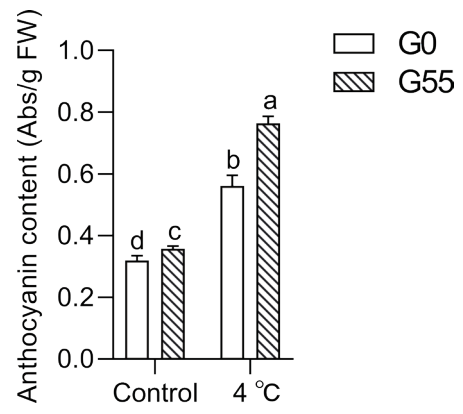

Supplement: Web_Material_uhae096 [file web_material_uhae096.zip › Fig.S6.pdf]

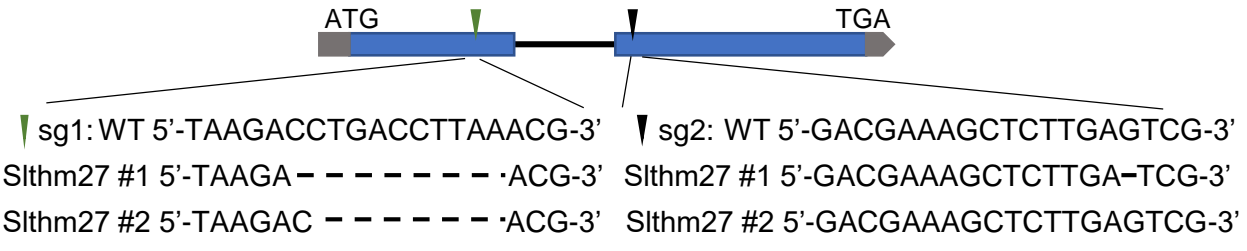

Supplement: Web_Material_uhae096 [file web_material_uhae096.zip › Fig.S11.pdf]

*SIGAD2*

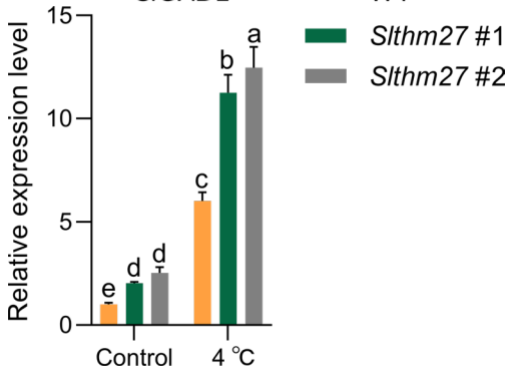

Supplement: Web_Material_uhae096 [file web_material_uhae096.zip › Fig.S12.pdf]
