# Supplementary material for: SlGAD2 is the target of SlTHM27, positively regulates cold tolerance by mediating anthocyanin biosynthesis in tomato
Source: Hortic Res. 2024 Apr 4;11(6):uhae096. doi: 10.1093/hr/uhae096 (PMC11161262; doi:10.1093/hr/uhae096)
Supplement: Web_Material_uhae096 [file web_material_uhae096.zip › Supplemental Figures.doc]

**Supplementary Figure**

**Fig. S1 Endogenous GABA content tomato leaves at designed time points of cold treatment.** Endogenous GABA content was measured by liquid chromatography-mass spectrometer (LC-MS). Values represent the average of four independent measurements, and error bars represent standard errors. Different letters above the columns indicate significant differences (P <0.05).

**Fig. S2 Multiple amino acid sequence alignment of SlGAD1-5.**

**Fig. S3 The relative expression levels of *SlGAD2* in transgenic tomatoes of overexpression *SlGAD2* (*SlGAD2* OE#4 and *SlGAD2* OE#5).** Values represent the average of three independent measurements, and error bars represent standard errors. Different letters above the columns indicate significant differences (*P* <0.05).

**Fig. S4 *SlGAD2* OE plants are more tolerant to cold stress.** (a) Phenotypic changes in WT and *SlGAD2* OE plants treated at low temperature (4°C) for 4 days. Bar, 2.5 cm.(b) Endogenous GABA content tomato leaves shown in (a). (c-e) Electrolyte leakage (c), MDA content (d) and H_2_O_2_ content (e) of *SlGAD2* OE plants under control and cold stress (4℃). Plants were exposed to cold stress for 4 days, and leaves were collected for an ion leakage assay, MDA and H_2_O_2_ content measurement. (f) NBT and DAB staining of leaves from WT and *SlGAD2*-overexpressing lines under control and cold stress (4℃). (g–i) SOD activity (g), POD activity (h) and CAT activity (i) in *SlGAD2* OE plants under control and cold stress. Plants were exposed to cold stress for 0 and 4 days, and leaves were collected for the measurements. Values represent the average of four independent measurements, and error bars represent standard errors. Different letters above the columns indicate significant differences (*P* <0.05).

**Fig. S5 *SlGAD2* induces anthocyanin synthesis in tomato seedlings.** (a) A large amount of pigment was accumulated near the leaf veins of *SlGAD2* OE plants under low-temperature treatment. Bar, 2 cm. (b) Anthocyanin extraction from *SlGAD2*-overexpressing lines (*SlGAD2* OE#4 and *SlGAD2* OE#5) and wild-type (WT) under control and cold stress (4℃). (c) Anthocyanin content of *SlGAD2* OE plants under control and cold stress (4℃). (d-g) Relative expression levels of *SlCHS*, *SlF3H*, *SlDFR* and *SlUFGT* genes under cold treatment in *SlGAD2* OE plants for 4 days. *SlACTIN* was used as an internal control. Values represent the average of four (c) or three (d-g) independent measurements, and error bars represent standard errors. Different letters above the columns indicate significant differences (*P* <0.05).

**Fig. S6 GABA induces anthocyanin accumulation under low-temperature stress.** (a) Exogenous spraying of 55 mM GABA stimulated endogenous GABA production in tomato. (b) Exogenous spraying of GABA significantly increased anthocyanin content of tomato leaves before and after cold treatment. Values represent the average of four independent measurements, and error bars represent standard errors. Different letters above the columns indicate significant differences (*P* <0.05).

**Fig. S7 CRISPR - Cas9 mediated target mutations in *SlGAD2*.** Schematic representation of the *SlGAD2* gene, with blue boxes and black lines indicating exons and introns, respectively, and gray areas representing the 5'UTR and 3'UTR. Green and orange triangles show CRISPR-Cas9 target sites. Each alignment between WT and mutant sequences containing the target site is shown below the schematic.

**Fig. S8 Multiple amino acid sequence alignment of SlTHM27 and MdMYB16.**

**Fig. S9 SlTHM27-MBP was not able to bind the ‘TTTGGT’ element on the *SlGAD2* promoter by EMSA analysis.**

**Fig. S10 Multi-alignment of the amino acid sequences of MYB type transcription repressors.** The NCBI accessions of MYB transcription factors are MdMYB16 (NM_001247046.1), AtMYB4 (NP_195574.1), ZmMYB31 (XM_035964451.1) and SlTHM27 (NM_001247046.1).

**Fig. S11 CRISPR - Cas9 mediated target mutations in *SlTHM27*.** Schematic representation of the *SlTHM27* gene, with blue boxes and black lines indicating exons and introns, respectively, and gray areas representing the 5'UTR and 3'UTR. Green and black triangles show CRISPR-Cas9 target sites. Each alignment between WT and mutant sequences containing the target site is shown below the schematic.

**Fig. S12 Relative expression level of *SlGAD2* in *Slthm27* mutant plants under control and cold stress for 4 days.** Values represent the average of three independent measurements, and error bars represent standard errors. Different letters above the columns indicate significant differences (*P* <0.05).

**Fig. S13** **Relative expression level of *SlGAD2* under silencing (pTRV-*SlGAD2*) or non-silencing *SlGAD2* (pTRV) conditions in WT and *Slthm27* mutant plants.** Statistical analysis was performed using a two-tailed Student’s *t-test* (*, *P* < 0.05; **, *P* < 0.01; ***, *P* < 0.001).
